# Research-Based Strength-Based Teaching and Support Strategies for Twice-Exceptional High School Students with Autism Spectrum Disorder

**DOI:** 10.3390/bs15060834

**Published:** 2025-06-19

**Authors:** Sally M. Reis, Sara J. Renzulli

**Affiliations:** Neag School of Education, University of Connecticut, Storrs, CT 06268, USA; sara.renzulli@uconn.edu

**Keywords:** social and emotional development, autism, academically talented, strength-based practices, counseling

## Abstract

In the last five years, several scholars have collaborated in an integrated research program focused on students identified with both academic talents and autism spectrum disorder (2eASD) with support from a Javits Gifted and Talented Students Education grant. Several different empirical studies were initiated and completed during this period, investigating the ways in which some of these twice-exceptional students have been able to be successful in secondary school and highly competitive colleges. In this article, we summarize findings from several of these studies, synthesizing implications and recommendations with a goal of offering research-based practices, especially related to healthy social and emotional development and strong academic achievement in students identified as 2eASD.

## 1. Introduction

Students across the world are being identified with autism spectrum disorder in increasing numbers, and among this group are many young people who are identified as both academically talented and with autism (2eASD). In the last five years, a research team conducted several studies to investigate the ways in which some of these twice-exceptional, neurodiverse students have emerged as academically successful in both secondary school and highly competitive colleges. We conducted qualitative case studies, interviews, surveys, and a comprehensive review of research to identify specific research-based practices related to how educators and parents can support both strong academic achievement as well as healthy social and emotional development in students identified as 2eASD. Of particular importance was the identification of strength-based practices for talented students, drawing upon the research of Renzulli and Reis (2014) and S. M. Baum et al. (2014) on enrichment practices and strength-based pedagogy.

## 2. Related Research

In this review of research, we present several pertinent recent research studies and their findings about challenges faced and successes achieved by this population of twice-exceptional students identified as 2eASD. A special focus is on the social and emotional development and support needed by these students to be academically and personally successful. The findings emerging from this review of recent research focusing on strategies that work are summarized in Table 1 below and discussed in greater detail.

### 2.1. Twice-Exceptionality and 2eASD

Individuals who exhibit gifted behaviors and/or academic talents and have also been diagnosed with one or more disabilities or disorders are referred to as twice exceptional, or 2e (Reis et al., 2014). These students usually need educational support for their gifts and talents to be developed, including enrichment and/or acceleration in their areas of talent and support for their specific social and emotional needs. They also need to receive services to compensate for and address their disabilities. Developed by a national Joint-Commission on Twice-Exceptional Learners, the following operational definition was used to identify students and refer to this population in the various research studies summarized in this article:

Twice-exceptional learners are students who demonstrate the potential for high achievement or creative productivity in one or more domains such as math, science, technology, the social arts, the visual, spatial, or performing arts or other areas of human productivity AND who manifest one or more disabilities as defined by federal or state eligibility criteria.

(Reis et al., 2014)

Increasing numbers of individuals with academic talents are being identified with autism spectrum disorder (ASD), a neurodevelopmental condition that is both complex and lifelong (Reis et al., 2014). Autism spectrum disorder is usually characterized by deficits in various adaptive behavior areas, including communication, socialization, and repetitive behaviors and/or interests (American Psychiatric Association, 2013). Individuals with 2eASD can exhibit behaviors, often associated with ASD, that may overlap with gifted behaviors, such as an intense focus on interests, often resulting in misidentifying one of these two exceptionalities. Some research has found that students with 2eASD often exhibit anxiety, difficulties with social interactions, and a special focus on special interests (Maskey et al., 2013; Reis et al., 1997, 2014, 2021, 2022a; Simonoff et al., 2008). Some of these interests may subsequently develop into lifelong passions. These characteristics may result in a failure to be identified as gifted. In other words, the talents of these students generally receive much less favorable attention than their deficits. And if the focus of their teachers and parents is deficit-based, they may fail to be identified as having talents or high potentials.

### 2.2. Recent Research About Students Identified as 2eASD

In our recent research, the first qualitative case study project completed as part of this systemic research program involved conducting interviews with 40 academically talented college students identified as 2eASD about their educational, social, and personal experiences contributing to their success in high school and subsequently in college (Madaus et al., 2022b; Madaus et al., 2022c; Reis et al., 2021, 2022a). Concurrently, a systematic review of the research literature on 2eASD individuals was also conducted (Gelbar et al., 2022). Additionally, a quantitative survey of 147 college accessibility personnel was conducted to probe their perceptions about factors that contribute to academic success in competitive colleges (Gelbar et al., 2022). Ten of these college disability service providers also participated in in-depth qualitative interviews (Madaus et al., 2022a), as did a sample of parents of the forty participants in the larger study (Madaus et al., 2022c). In addition, in another qualitative study, a representative sample of high school teachers and school counselors were interviewed about their experiences teaching and working with students who are 2E-ASD (Austermann et al., 2025; Renzulli & Austermann, in press; Reis et al., 2025). In addition, our researchers developed an online summer program for 22 high school students identified as 2E-ASD. Using qualitative methods, these participants were subsequently interviewed soon after they entered college to investigate the factors that most contributed to their success (Austermann et al., 2023). This collective body of research focused on factors contributing to the academic and social/emotional success of this group of students, identifying important factors reported. Academic strategies that contributed to success included interest-based opportunities often focused on interests as well as participation in enrichment opportunities such as advanced and honors courses, camps based on interests, and interest-based extra-curricular activities. In addition, various social emotional challenges faced by these young adults were also identified across this research, such as persistent and high levels of anxiety, isolation, and feelings of being different as the leading challenges requiring support. Findings from this integrated body of research also identified specific social and emotional challenges facing students with 2eASD, including problems with peer relationships, difficulty establishing relationships with teachers, social struggles, complications with self-advocacy, struggles with transitions, and mental health challenges, especially anxiety. These various challenges are described and discussed in more depth in this article.

Other recent research works about strength-based learning have also focused on the need for a combination of academic rigor, challenge, interest-based work, and engagement to improve academic achievement for students identified as 2e (S. M. Baum et al., 2014, 2017; S. Baum & Olenchak, 2022; Josephson et al., 2018; Foley-Nicpon et al., 2013; Reis et al., 2014). Our recent research summarized in this article (Madaus et al., 2022b, 2022c; Reis et al., 2021, 2022a) also supports the use of advanced and rigorous content, interest-based enrichment, and strength-based strategies to increase and enhance the academic success of exceptional students with 2eASD. While research about the use of specific strategies is sparse, other corresponding research works concur about the importance of hands-on activities for students with ASD (Danker et al., 2019; Hillier et al., 2021). Current researchers believe that strength-based strategies for this population should be individualized, although most do not define the meaning of strength-based learning (Dipeolu et al., 2015; Hillier et al., 2021; Holcombe & Plunkett, 2016; Saggers, 2015; White et al., 2017). Other recent research works support findings from our recent work, advocating for the use of strength-based approaches for 2e students that simultaneously accommodate students’ deficits or weaknesses (S. M. Baum et al., 2014, 2017; S. Baum & Olenchak, 2022; Foley-Nicpon et al., 2013; Reis et al., 2014). Focusing on strengths can increase student engagement while also reducing problem behaviors by reinforcing positive social experiences (S. M. Baum et al., 2014; Dipeolu et al., 2015), often experienced during their involvement in extracurricular activities that support their interests and academic strengths (Reis et al., 2021, 2022b). These opportunities enable twice-exceptional individuals to develop their interests, experience positive social connections, hone their executive functioning skills, and further explore their talents and interests.

### 2.3. Social and Emotional Development in Students with 2eASD

Students identified as 2eASD across our recent research faced challenges with their social and emotional development (Gelbar et al., 2022; Madaus et al., 2022a, 2022b; Reis et al., 2021, 2022a). For example, all parents we interviewed reported that their children experienced feelings of social isolation and loneliness at various times during elementary and high school years. Our participants, according to their parents, also experienced challenges with emotional regulation and difficulties dealing with high levels of sensory sensitivities. Other areas of social and emotional concern discussed by our participants included social struggles and an absence of peer interactions, often due to different types of social challenges, such as not knowing how to make a joke, not being able to respond quickly to social situations, or not being able to participate in conversations. Some participants explained that they had been grouped with other students identified as needing special education who had experienced emotional and behavioral difficulties, often severe, when they were in elementary school and secondary school. When this occurred, the students identified as 2eASD often imitated inappropriate behaviors and struggled with negative peer influences, especially with not having models of other academically talented students without ASD, and accordingly, generally without behavioral challenges. Many of our participants explained that they had few friends in elementary school and middle school and were initially uncomfortable in most, if not all, social situations and settings. On the positive side, these highly academically successful students also discussed how their parents and teachers helped them gradually learn to develop healthy social and emotional habits of mind (Madaus et al., 2022b, 2022c; Reis et al., 2021, 2022a). For example, teachers and counselors often provided safe spaces for them in school and discussed how to handle missed social cues and overcome associated problems with other social situations (Renzulli & Austermann, in press). Some of their teachers and counselors used role modeling to demonstrate ways to interact in new social settings (Renzulli & Austermann, in press). Proactive strategies for positive social and emotional development were consistently discussed across various research studies by these successful high school educators and college students identified as 2eASD. These strategies include, for 2eASD students, how to handle stressful situations to achieve positive outcomes and for students without ASD how to interact socially and across educational and extra-curricular activities with students identified on the ASD spectrum.

### 2.4. Anxiety

Research conducted by our group during the last few years (Gelbar et al., 2022; Madaus et al., 2022a, 2022b, 2022c; Reis et al., 2021, 2022a) found a high prevalence of anxiety in all participants with 2eASD, contributing to challenges to their academic and personal success. These challenges usually focused on various risk factors contributing to anxiety in this population, such as the likelihood of dropping out of college or quitting an extra-curricular activity or honors or advanced class. In one meta-analysis focusing on research about anxiety in children with ASD, researchers found that rates of anxiety and general anxiety disorder (GAD) were higher among older children and separation anxiety and obsessive/compulsive disorder (OCD) were more common among younger children (Van Steensel et al., 2011). Adolescents with ASD have higher levels of anxiety than their peers without ASD, and higher functioning individuals with ASD have been found to experience a higher rate of anxiety disorders than those with other types of disabilities and students without ASD (Gillott et al., 2001; Kim et al., 2000; Muris et al., 2011). In a study of 108 high-functioning children with ASD enrolled in treatment trials for anxiety, almost all (91.6%) met the criteria for two or more anxiety disorders, especially social phobia (41.7%) and generalized anxiety disorder (25.9%; Ung et al., 2015). Collectively, across our research (Madaus et al., 2022b, 2022c; Reis et al., 2021, 2022a), anxiety disorders were prevalent in the vast majority of our identified 2eASD participants.

### 2.5. Peer and Social Relationships

Another critical factor influencing the success of students identified as 2eASD in school is their relationships with peers. For these students, relationships are extremely important and can enhance or, in some cases, negatively affect their school experience. Students with ASD report the importance of positive peer experiences in building relationships both during and after high school (Bottema-Beutel et al., 2019). Conversely, negative school experiences are also related to social relationships with others, with students with ASD confronted with bullying, loneliness, and feelings of being different (Bottema-Beutel et al., 2019; Saggers, 2015). These challenging social experiences have led students with ASD to isolate themselves, creating a vacuum of social support and even higher levels of anxiety (Koegel et al., 2013). Our research had similar findings as our participants struggled with social relationships with other students with and without ASD (Reis et al., 2022a). The teachers and counselors we interviewed, however, were aware of these challenges and discussed the importance of working to build peer relationships and helping students with 2eASD to establish relationships with students who share their interests and talents. They also worked diligently to help our participants minimize their feelings of isolation by encouraging them to participate in honors classes, clubs, sports, and other extra-curricular activities in areas of high interest (Austermann et al., 2025; Reis et al., 2021, 2022a; Renzulli & Austermann, in press).

Unfortunately, many 2e-ASD students who attend college do not experience the same success as students without disabilities for a variety of reasons, including social isolation and feelings of loneliness. A student’s sense of belonging can positively influence students’ persistence in college (Hausmann et al., 2007), as well as their self-advocacy, social relationships, and personal beliefs about living with ASD. Students with 2e-ASD face challenges in these areas, and providing opportunities to foster their strengths and interests in honors classes and enrichment programs can result in improved self-awareness, social relationships, and academic success for 2e-ASD students (Reis et al., 2021, 2022a). Our participants discussed their acquisition of skills helping them learn to interact appropriately with adults and other peers, usually due to experiences of varied enrichment opportunities and building relationships in high school through advanced classes, projects, and extracurricular activities (Reis et al., 2022b). When they were able to build relationships and participate in activities that address their social anxiety, these young adults with 2eASD were able to understand the importance of these relationships over time. For example, our participants often report struggling during their early years of high school as well, having zero or few friends, suffering extreme social anxiety, and understanding the ways in which they had to work on developing friendships and relationships. And as these are challenging areas for many 2eASD students, our research demonstrated that their teachers and counselors worked diligently to involve these students in clubs and activities based on their interests. These experiences helped them to develop some new friendships with other students with and without ASD (Renzulli & Austermann, in press). Students in our studies also discussed the ways that their academic peers without ASD accepted them and interacted with them during the time in which they were involved in interest-based extra-curricular activities.

Our participants learned from previous negative mistakes as well as positive experiences, and eventually, most learned to understand when it was time to be alone and recharge their social batteries, as well as when they needed to pursue social interactions, avoiding what is sometimes referred to as autistic overwhelm. Developing appropriate cues and higher levels of social awareness required them to learn how to create and maintain appropriate social connections in both high school and college and to help non-ASD peers understand that normal social teen interactions are generally not enjoyable for individuals with ASD. For some, relying on a high school network of friends that they could contact and access online provided a safety net when they began college. This enabled them to rely on these previous relationships and reduced their anxiety about making new friends and relationships as they began college. In summary, most of the participants in our study, were able to develop an emotionally safe community, in and out of the classroom, that was important for their social and academic development both in high school and college.

In summary, the students we studied with 2eASD experienced many challenges with understanding and reacting appropriately to social cues and interactions due to their many challenges with communication and anxiety, but they found ways to address these challenges, including developing relationships with peers (Reis et al., 2021, 2022b). Our participants came to understand that successful peer and adult relationships enhanced and contributed to their successful school experiences. Reflecting on high school, they understood that their positive social experiences helped to prepare them for a successful academic future in competitive colleges. The negative experiences and social challenges they experienced earlier in school, including bullying, loneliness, periods of isolation, and feeling different, initially resulted in anxiety about college and a tendency to isolate themselves socially, but they all found ways to focus on more positive experiences, such as friends that they made, social experiences that were enjoyable, and challenging content classes in which they made acquaintances and friends. Interestingly, some 2eASD students in our study reported having a few friends but deciding not to spend a great deal of time with them due to their limited ability to socialize (Austermann et al., 2023; Reis et al., 2021, 2022a). Often, the choice not to engage socially related to negative experiences from previous difficulties with conversations and awkwardness in social settings, a common struggle for students with ASD. Teachers and counselors that we studied understood the importance of peer relationships for these students with 2eASD, noting that students with ASD who can establish relationships with other students can minimize feelings of isolation (Austermann et al., 2023; Renzulli & Austermann, in press).

### 2.6. Self-Determination

Self-determination is the ability to make life decisions independently (Wehmeyer, 2015), and educators often focus on developing self-determination in their students, particularly those with disabilities, as it helps to improve outcomes in life (Wehmeyer & Schalock, 2001). When students identified as 2eASD increase their self-determination, they display more choice in selecting education and career paths that align with their interests and strengths. They can also strive to create positive academic and social environments in high school and college. In our research with 40 successful 2e-ASD college students, the majority experienced both academic and personal barriers to success in college. The academic barriers included challenges with specific content skills in areas such as writing and oral discussions. Their non-academic barriers included mental health concerns, anxiety, organizational and time management skills, motivation or interest in a subject, and social and emotional development. Several opportunities positively contributed to increased self-determination including summer programs and residential camps (Reis et al., 2021, 2022b). According to our participants, involvement in one or more extracurricular activities in high school contributed to their self-determination. Our findings also support time spent engaging with neurotypical peers in their preferred interest areas, often time either in competitive advanced classes or interest-based extra-curricular activities (Reis et al., 2021). Most of our sample found friends and developed self-determination through sports, gaming clubs, and other extra-curricular activities.

### 2.7. Post-Secondary Institutions, Areas of Study, and Important Experiences

Participants also discussed the colleges and universities they attended, or hoped to attend, as well as their intended areas of study. Some students purposefully applied to or planned to attend a college or university close to home, as they believed living at and commuting from home would be helpful for their successful college transition. Over half of our participants reported interest in at least one competitive college or university, suggesting that students who are 2eASD can attend and excel at competitive institutions (Reis et al., 2021, 2022a). Some students also reported that they considered the academic programs offered and the resources and support available when considering their school, reflecting their understanding of being 2e, as suggested by other research works in this area (S. M. Baum et al., 2014; Foley-Nicpon et al., 2013).

An important finding from this study was that students believed they were prepared for their transition to college because of their advanced learning opportunities in high school but also demonstrated a strong understanding of their dual exceptionalities. These findings support previous work by researchers in this field (e.g., S. M. Baum et al., 2014; Foley-Nicpon et al., 2013; Morrison & Rizza, 2007). This self-knowledge suggests that students with 2eASD understand their unique needs, including opportunities to develop their talents and interests but also ways to accommodate their special education needs.

In addition to an understanding of 2e, another finding of this study that emerged as important for college transition and preparation is related to the need for appropriate adult support. All successful students in our research could identify one or two adults they felt connected to in high school, such as a teacher, counselor, or parent. They sought support from their counselors, teachers, and parents in their college-transition process (Madaus et al., 2022c; Renzulli & Austermann, in press). Although participants in our studies believed they were supported in their interest and strength areas in high school, they also expressed concerns related to managing their anxieties time, social lives, and workload. They also often experienced frustrations with task completion and communication and often reported feeling overwhelmed with writing tasks.

## 3. Summary

The findings that emerged from our research about highly successful college students focused on their experiences in high school and college that led us to better understand which strategies teachers and counselors used with this population of students to help them be academically successful.

Our research identified the importance of developing strength-based content expertise and interests in 2e-ASD students as well as having them participate in enrichment opportunities including advanced courses, overnight camps, and extra-curricular activities. Most of these students were able to identify what made a positive difference in their high school success, such as being told they had academic talents, participating in challenging honors and advanced placement classes, and being given specific enrichment opportunities such as the ability to complete independent projects with a mentor in their areas of interest. They also discussed the importance of interest-based extra-curricular activities, often after having several different experiences and some failures before they found the right club or sport. Many of these students chose to participate in individual sports such as track. About half also discussed their experiences at residential summer programs, often based on a strength or interest, such as nature or the arts. Many also pursued other advanced educational experiences to help develop their talents. A specific focus on and plan for talent development was critically important for their academic success. One unanticipated outcome from this research was the importance of advanced content experiences for competitive college preparation and the importance of having teachers and counselors participate in recommending these classes (Reis et al., 2021, 2022a). Related to the social and emotional development of this group, we found that strong and positive relationships with teachers and counselors made a positive difference and provided a safe place both physically and emotionally for them (Austermann et al., 2023; Renzulli & Austermann, in press). We also found that these 2eASD students can learn how to overcome anxiety while building social connections in various advanced classes and extra-curricular activities based on interests.

Another finding from our systematic review of previous research related to 2eASD individuals showed how little empirical research had been published on students identified as 2eASD (Gelbar et al., 2022). These students have recently emerged as a focus of research, as the numbers of individuals identified are increasing. However, only 32 articles were published between 1996 and 2019, and of these 32 articles, 62.5% were data-based, whereas the remaining 37.5% were review or conceptual articles. Little research is being conducted on this population. Some of the research conducted recently involved case studies; others were correlational in nature, and most were descriptive, focusing on participants’ characteristics and how they were identified. A wide range of definitions were utilized in the literature, and when our project began, there was no empirical research that had been published about this population.

As part of our research related to 40 students with 2eASD who attended highly competitive colleges, we explored the personal perceptions and institutional factors that facilitated academic success, as well as challenges encountered, in college and university. Interesting findings from the study included that most of our participants had excellent reading and some had excellent writing skills but struggled with mathematics, which was unexpected as popular folklore about these students suggests that many excel in STEM areas. When asked about the personal trait that most enabled them to be successful in college, 26 of the 40 participants described a driving passion for learning. Half of the participants were motivated to succeed academically by the independence college offers, as well as the flexibility to take advantage of a range of opportunities, and the development of their personal autonomy. Many craved the independence of college and university life but also needed some support from their families. Most participants believed that current college and university faculty members’ instructional practices were barriers to learning, describing long lectures and a lack of engaging active learning opportunities. They also lamented not being provided with lecture notes and power point slides, which would have made their learning more efficient. However, despite these challenges, they were succeeding at competitive universities and colleges.

## 4. Implications

Findings from these collective studies demonstrate that students identified with 2eASD have dreams to apply and matriculate, as well as succeed, in competitive colleges and universities. Educators and parents involved in supporting these students should consider several implications based on this research. Parents and school staff should advocate for extracurricular involvement and enrichment opportunities for students who are 2eASD (Reis et al., 2021, 2022b). The participants in this study attributed some of their social successes in college to their extracurricular involvement in high school. Most of those involved in this study prefer social activities related to technology, games, and other extracurricular opportunities in areas of interest so educators should consider offering these opportunities and supporting them as these students develop an extracurricular or enrichment activity in their interest area. This will help to prepare them for future involvement in an area of their interest, while also developing relationships with peers and adults.

Opportunities to enroll in summer programs also prepare some students who are 2eASD for college transition (Austermann et al., 2023). Educators who work with these students should realize that summer opportunities related to students’ interests and strengths can help develop students’ college readiness. These opportunities, which are available to these talented students, may be day programs at college universities, online opportunities, or ideally residential programs, which may offer added benefits of developing social and independent living skills.

Parents and educators should consider recommending competitive colleges as reasonable options when these academically talented students begin their college transition planning. Students with academic strengths should attend universities that fit their academic needs and meet their career aspirations. With supports and recommendations such as those mentioned in this article, students who are 2eASD can learn the skills necessary to succeed at competitive colleges and universities. Although some students prefer to attend a post-secondary institution close to home, as the opportunity to remain at home with family support helped with their transition, competitive four-year institutions should not be ruled out, and the support and resources offered at these institutions should be considered for this population (Madaus et al., 2022c; Reis et al., 2021, 2022a).

Teachers and school counselors should explicitly help students who are 2eASD to develop ways to address and manage their anxiety. As these students generally experience anxiety more intensely and more often than their typically developing peers, teachers and counselors should help them learn to manage their anxiety by identifying triggers and recognizing how to handle feelings related to anxiety (Renzulli & Austermann, in press). Students with 2eASD may have a difficult time learning executive function skills as well as advanced study skills, and these are necessary skills for students who are 2eASD, who are taking advanced-level academics and aspiring to apply to competitive colleges and universities (Madaus et al., 2022b).

## Figures and Tables

**Table 1:** Academic and social/emotional success strategies reported by 2eASD students.

**1.** **Focus actively on Identifying Strengths and Interests** Use instruments or experiences to identify strengths and interests Focus on strengths to increase engagement Use interests and strengths to reinforce positive social experiences
**2.** **Experience Interest-based Academic Opportunities** Participate in advanced and honors courses Attend residential and day camps and programs based on interests Engage in carefully selected extra-curricular activities
**3.** **Experience and Engagement in Appropriate Academic Rigor** Challenging content Differentiated instruction to accommodate students’ deficits or weaknesses Active learning, such as engaging in interest-based projects
**4.** **Translate interest and Strength-based opportunities** Actively develop interests Experience positive social connections Improve executive functioning skills Explore majors and careers related to interests Focus on cultivating talents
**5.** **Develop Positive Social and Emotional Growth** Develop positive emotional regulation Learn and practice healthy social and emotional habits of mind Turn stressful situations into positive outcomes Establish new relationships with peers (both with and without ASD) Interact socially in educational and extra-curricular activities with others Learn from positive peer influences
**6.** **Facing challenges and Finding Solutions to Social Issues** Learn from and interacting with new peers Watch and imitating positive peer behaviors Minimize isolation Try and evaluate individual ways to address anxiety
**7.** **Build Relationships with Caring Adults** Learn to ask for and accept help Understand how to interact with adults such as teachers and counselors
**8.** **Prepare for Transitions to College** Use a multifaceted approach to selecting college focused on academic and social needs Develop, personalize, and improve executive functioning skills Understand and participate in various support services at the college level Create a personalized emotionally safe community

## Data Availability

No new data were created or analyzed in this study. Data sharing is not applicable to this article.
